# Solid-State Transformation of an Additive Manufactured Inconel 625 Alloy at 700 °C

**DOI:** 10.3390/app11188643

**Published:** 2021

**Authors:** Fan Zhang, Jan Ilavsky, Greta Lindwall, Mark R. Stoudt, Lyle E. Levine, Andrew J. Allen

**Affiliations:** 1 Material Measurement Laboratory, National Institute of Standards and Technology, Gaithersburg, MD 20899, USA;; 2 X-ray Science Division, Advanced Photon Source, Argonne National Laboratory, Argonne, IL 60559, USA;; 3 KTH Royal Institute of Technology, Brinellvägen 23, SE-10044 Stockholm, Sweden;

**Keywords:** additive manufacturing, nickel-based superalloy, phase evolution, synchrotron, small-angle X-ray scattering, in situ diffraction, CALPHAD

## Abstract

Inconel 625, a nickel-based superalloy, has drawn much attention in the emerging field of additive manufacturing (AM) because of its excellent weldability and resistance to hot cracking. The extreme processing condition of AM often introduces enormous residual stress (hundreds of MPa to GPa) in the as-fabricated parts, which requires stress-relief heat treatment to remove or reduce the internal stresses. Typical residual stress heat treatment for AM Inconel 625, conducted at 800 °C or 870 °C, introduces a substantial precipitation of the δ phase, a deleterious intermetallic phase. In this work, we used synchrotron-based in situ scattering and diffraction methods and ex situ electron microscopy to investigate the solid-state transformation of an AM Inconel 625 at 700 °C. Our results show that while the δ phase still precipitates from the matrix at this temperature, its precipitation rate and size at a given time are both smaller when compared with their counterparts during typical heat treatment temperatures of 800 °C and 870 °C. A comparison with thermodynamic modeling predictions elucidates these experimental findings. Our work provides the rigorous microstructural kinetics data required to explore the feasibility of a promising lower-temperature stress-relief heat treatment for AM Inconel 625. The combined methodology is readily extendable to investigate the solid-state transformation of other AM alloys.

## 1. Introduction

Inconel 625 (IN625) is a nickel-based solid-solution superalloy with a Ni-Cr matrix strengthened by Nb/Mo solutes [1]. IN625 features high strength, high fracture toughness, and good corrosion resistance and finds many applications in marine and energy industries, for example, turbine engine components, fuel and exhaust systems, and chemical processing components. IN625 also has excellent weldability and resistance to hot cracking. These characteristics make IN625 a primary alloy in the recent advancement of various additive manufacturing (AM) technologies [2–7], where only a few existing alloys out of more than 5500 alloys in use today meet the stringent printability criteria imposed by AM [8].

Printability represents an inherent and fundamental challenge to AM. One central issue related to this challenge is the build-up of residual stress during the rapid solidification and subsequent thermal cycles with localized cooling rates as high as 1 × 10^6^ °C/s to 1 × 10^7^ °C/s [9]. For example, neutron diffraction measurements on AM IN625 have demonstrated that within a single component, the residual stress variation can be as significant as 1 GPa [6,10]. Residual stresses of this magnitude can lead to part distortion, introduce fatal defects, and adversely affect the fabricated part’s mechanical properties and performance [11,12]. While several strategies have been developed to reduce the residual stress introduced during the fabrication processes, such as optimizing the scan pattern [13,14] or heating the base plate [15], stress-relief heat treatments still represent the most common and reliable approach to mitigating residual stress.

Another ubiquitous phenomenon associated with AM is microsegregation [16,17]. In conventional manufacturing processes, macrosegregation manifests as compositional variations on length scales ranging from millimeters to centimeters or even meters [18]. The finite size of the melt pool in AM creates much more localized microsegregation, mainly due to the difference in the solubility of alloying elements in the liquid phase and solid matrix phase. In nickel-based superalloys such as IN625, microsegregation leads to a high concentration of refractory elements, for instance, Mo and Nb, near the interdendritic regions [19]. A distribution coefficient *k*, defined as the mass-concentration ratio between those of the dendrite center and the interdendritic region, describes the degree of elemental segregation. In IN625 welds, the *k* values for Mo and Nb are typically 0.95 and 0.50, respectively [20]. In AM IN625 fabricated using powder laser-bed fusion (PLB-F), thermodynamic simulations predicted the *k* values for Mo and Nb to be approximately 0.3 and 0.1, respectively [19]. In other words, AM fabrication can lead to a more localized and more extreme elemental segregation when compared with the traditional welding processes.

The need to relieve residual stress and the presence of microsegregation can generate an unfavorable situation for microstructural control and optimization. AM IN625 serves as a good example because it has local compositions well outside the standard compositional range for IN625, rendering the as-fabricated part not being IN625 everywhere despite both the powder composition and the average nominal composition being within the standard [21]. A stress-relief heat treatment at 870 °C for one hour, as recommended by the AM machine manufacturer [22], is highly effective in relieving the residual stress. However, it also introduces a significant amount of large δ phase precipitates, which is a phase that negatively affects the performance of IN625. An alternative stress-relief heat treatment at 800 °C for two hours proves effective in reducing the residual stress, too. However, it still creates sizable δ phase precipitates with major dimension exceeding 600 nm. A separate strategy is to completely remove the microsegregation using a high-temperature homogenization heat treatment. For example, a heat treatment at 1150 °C for one hour completely homogenizes the alloy. However, this heat treatment promotes grain growth and can be both challenging and costly to implement for industrial-scale large parts due to the time required for the temperature to equilibrate as well as the high annealing temperature required.

These complicating factors contribute to an industrial need to investigate the feasibility of using lower temperature stress-relief heat treatments. To understand the microstructural responses of AM IN625, in this study, we investigate the solid-state transformation kinetics of an AM IN625 alloy at 700 °C primarily using synchrotron-based in situ scattering and diffraction methods. Specifically, we use X-ray diffraction to monitor the phase transformation kinetics and small-angle X-ray scattering to evaluate the morphological changes in the precipitates. In contrast to most studies of the effect of heat treatment on nickel-based superalloys, where experimental evidence is mainly gathered from microscopy and from in-house X-ray diffraction data, synchrotron measurements probe a fixed and significantly larger sample volume through in situ experiments that allow the annealing kinetics to be unambiguously determined. Such results are also more statistically representative. The kinetics results from the same sample volume are elucidated with thermodynamic predictions by CALPHAD (Computer Coupling of Phase Diagrams and Thermochemistry) methods.

## 2. Materials and Methods

### 2.1. Material Fabrication and Sample Preparation

The Measurement Science for Additive Manufacturing Program at the National Institute of Standards and Technology (NIST), U.S.A., fabricated 15 mm cubes of IN625 using an EOS INT M270 laser powder-bed fusion (L-PBF) instrument (EOS GmbH, Munich, Germany). The virgin IN625 powders, procured from EOS, have a composition that is within the allowed composition range specified by the ASTM Standard for Additive Manufacturing Nickel Alloy UNS N06625. The vendor-supplied compositions are listed in Table 1. The fabrication parameters include a Nd:YAG laser operated at 195 W, a scanning speed at 800 mm/s, and a hatch spacing of 100 μm. During the fabrication, the melt-pool width varied between 105 and 115 μm. More details about the fabrication can be found elsewhere [19].

### 2.2. Ex Situ Scanning Electron Microscopy (SEM)

We used scanning electron microscopy (SEM) to perform ex situ microstructural examinations of the as-fabricated and heat-treated samples. The JEOL S-7100F (JEOL, Ltd., Akishima, Tokyo, Japan) field emission SEM is equipped with an Oxford X-MAXN (Oxford Instruments Plc., Abingdon, UK) energy-dispersive X-ray spectrometry (EDS) detector. We operated the SEM at 15 kV.

To evaluate the effect of heat treatment on the microstructure of IN625, we encapsulated IN625 specimens in evacuated ampoules and performed heat treatments at 700 °C and 800 °C. We polished the SEM specimens following standard metallographic procedures, etched the surface with aqua regia, and performed the microstructural analysis with SEM. For this characterization, the imaged sample surfaces are parallel to the build direction, allowing microstructural information of the dendritic and interdendritic regions to be captured.

### 2.3. In Situ Synchrotron Small Angle X-ray Scattering and X-ray Diffraction

We performed synchrotron-based, in situ ultra-small-angle X-ray scattering (USAXS), small-angle X-ray scattering (SAXS), and XRD measurements at the USAXS facility at the Advanced Photon Source, Argonne National Laboratory, U.S.A. [23]. The in situ USAXS and SAXS monitor the morphology changes during a solid-state transformation induced by heat treatment. Within its detection limits, the in situ XRD provides information regarding the nature of the solid-state transformation. Combined, USAXS, SAXS, and XRD cover a continuous scattering *q* range from 1 × 10^−4^ Å^−1^ to ≈6.5 Å^−1^. Here, *q* = 4π/*λ* sin(*θ*), where *λ* is the X-ray wavelength and *θ* is one-half of the scattering angle *2θ*. More details about this setup can be found elsewhere [24].

For this study, we used monochromatic X-rays at 21 keV (*λ* = 0.5904 Å). The X-ray flux density at the sample is in the order of 10^13^ mm^−2^ s^−1^. The as-fabricated sample was mechanically polished to ≈50 μm in thickness. We used a Linkam 1500 thermal stage to control the temperature. After an initial measurement at room temperature, we performed a 10.5 h isothermal hold at 700 °C, with a heating rate from room temperature to the target temperature at 200 °C per min. The data acquisition times for USAXS, SAXS, and XRD are 90 s, 30 s, and 60 s, respectively, leading to a measurement time resolution of ≈5 min. The spatial dimensions of the gauge volume area were 0.8 mm × 0.8 mm for USAXS and 0.8 mm × 0.2 mm for SAXS and XRD.

### 2.4. Thermodynamic Calculations

In this work, we performed thermodynamic calculations using the CALPHAD software Thermo-Calc and its related thermodynamic database for Ni-based systems, TCNI9/Ni superalloys [25,26]. To compare to the experimentally observed precipitation events, we calculated the precipitation kinetics using the TC-PRISMA module [27–29]. This module is based on the Langer–Schwartz theory [30] and Kampmann–Wagner numerical methods [31,32] and calculates the nucleation, growth, and coarsening of precipitates in a multicomponent and multiphase system by integrating thermodynamic and diffusion information provided by CALPHAD descriptions. The simulation output includes the time-dependent evolution of the particle size distribution, number density, mean radius, and volume fraction. More details about the CALPHAD calculations can be found elsewhere [33].

## 3. Results and Discussion

**Figure 1.**
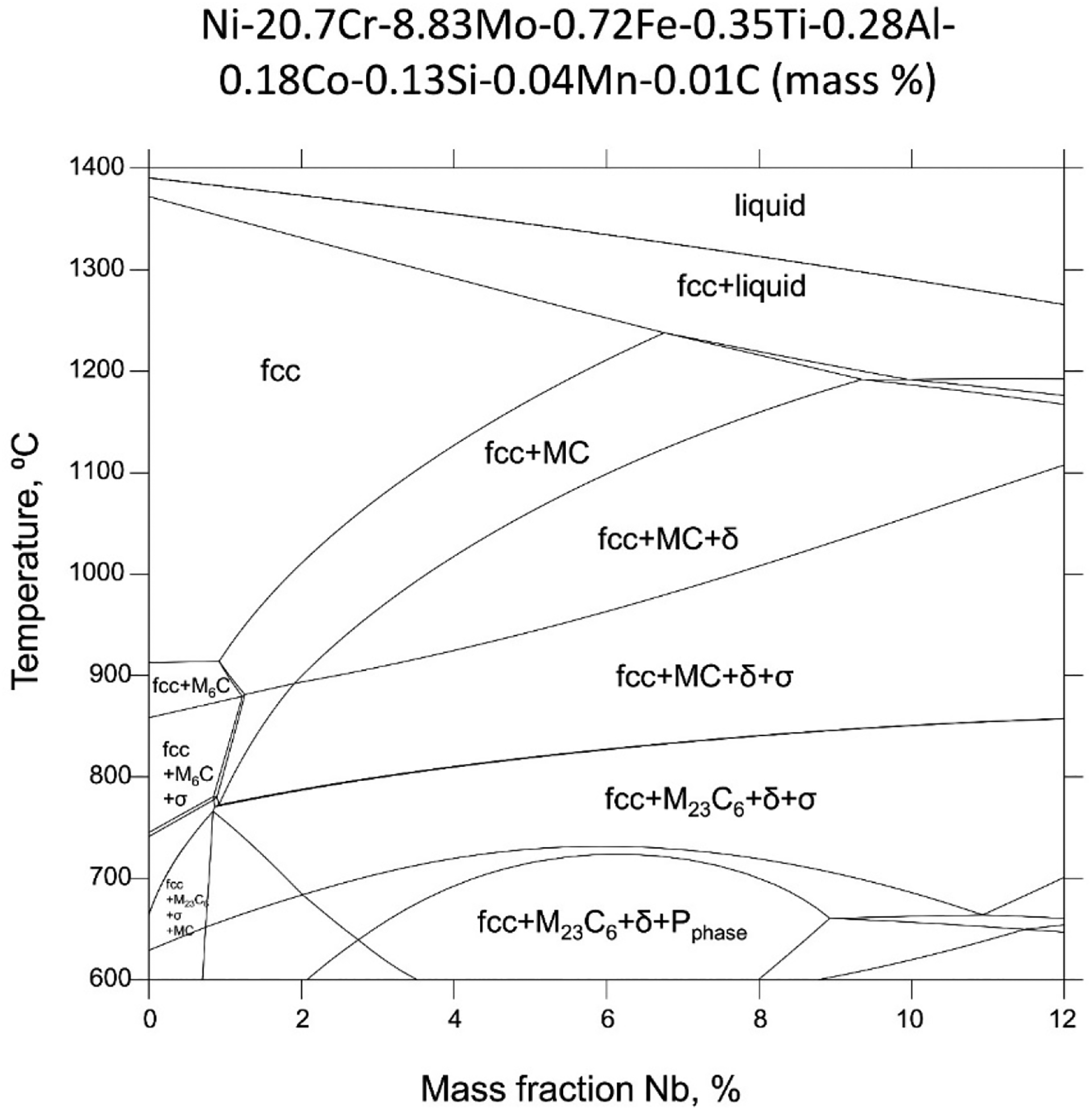
A steady-state Nb isopleth section of IN625 phase diagram constructed assuming a composition of Ni-20.7Cr-8.83Mo-0.72Fe-0.35Ti-0.28Al-0.18Co-0.13Si-0.04Mn-0.01C (mass%).

Figure 1 shows an equilibrium Nb-isopleth for the powder composition listed in Table 1. In addition to the FCC matrix, MC, M_23_C_6_, *σ*, *P*, and *δ* are thermodynamically stable equilibrium phases. δ, especially, is stable over a wide temperature range from below 600 to ≈1200 °C, depending on the mass fraction of Nb. We have previously established that significant microsegregation in the interdendritic region exists in the as-fabricated IN625 due to solute rejection caused by the difference in solubility in liquid and solid phases [19,34]. CALPHAD-based solidification simulations predicted by the Scheil–Gulliver model and by DICTRA using finite-element-analysis thermal-model predictions as input suggests extreme microsegregations of alloying elements of Mo and Nb. For example, the predicted Nb mass fraction ranges from ≈2% to ≈22% between secondary dendritic cores, which is well beyond the allowable range of Nb of between 3.15% and 4.15% (Table 1). Previous synchrotron SAXS measurements demonstrated that the microsegregation is concentrated near the interdendritic centers on a scale of 10 nm [35], which is consistent with model predictions [19]. This type of extreme microsegregation effectively renders the as-fabricated IN625 part not within the spec of IN625 in all places, resulting in unintended and deleterious solid-state transformations in this alloy.

**Figure 2.**
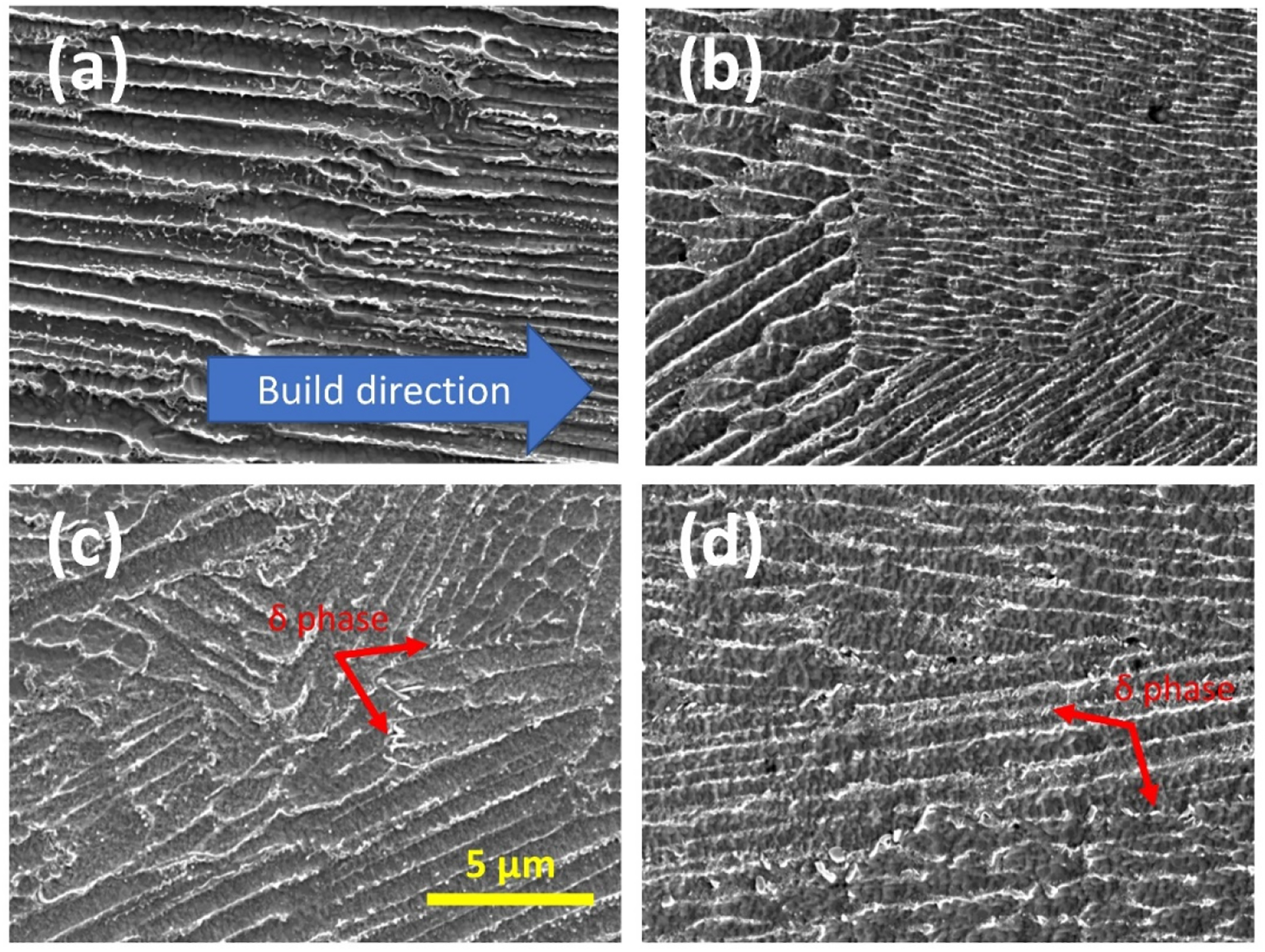
Microstructure in AM IN625 under four different conditions (**a**) as-fabricated, (**b**) after a one-hour heat treatment at 700 °C, (**c**) after a 24 h heat treatment at 700 °C, and (**d**) after a one-hour heat treatment at 800 °C. The imaged surfaces are all parallel to the build direction. Red arrows in (**c**,**d**) highlight the platelet δ phase precipitates.

Figure 2 shows the SEM images of AM IN625 under four different conditions (Figure 2a, as-fabricated, Figure 2b, one hour at 700 °C, Figure 2c, 24 h at 700 °C, and Figure 2d, one hour at 800 °C), with the imaged surfaces parallel to the build direction. The dendritic microstructure is visible in all four images. An EDS analysis of the as-fabricated specimen reveals that the interdendritic regions are enriched in Nb and Mo, and the dendritic regions are enriched in Ni and Cr. The effects of different heat treatment conditions on the microstructures are subtle, with one-hour heat treatment at 700 °C leading to no visually observable differences under the measurement conditions. In contrast, a prolonged heat treatment at 700 °C promotes the formation of precipitates of a platelet morphology near the interdendritic regions. The morphology of this phase is consistent with previous observation of δ phase, with the nucleation of the δ phase being more favorable with a higher concentration of Nb and Mo [33].

A one-hour heat treatment at 800 °C leads to a similar change to the microstructure with the formation of δ phase precipitates, as observed in Figure 2c (24 h at 700 °C). We note that in both Figure 2c,d, the δ precipitates have comparable sizes. The difference in the heat treatment duration suggests that the precipitation kinetics of the δ phase precipitates is much accelerated at 800 °C, compared with 700 °C, which is consistent with the TTT diagrams [33,36] previously constructed for AM IN625. This slowdown in precipitate growth can be significant for residual-stress relief heat treatment. Previous neutron diffraction residual stress experiments demonstrated that one-hour heat treatment at 870 °C [6] and two-hour heat treatment at 800 °C [10] could effectively reduce residual stresses to less than 13% of the initial, as-fabricated levels. However, heat treatment at these temperatures creates a favorable thermodynamic condition for the precipitation of the δ phase precipitates. In both cases (one hour at 870 °C and two hours at 800 °C), the major dimension of the δ phase precipitates has a comparable nominal size of ≈500 nm [21,24]. These large precipitates preferentially grow in the interdendritic regions and decrease the ductility, fracture toughness, and the corrosion resistance of IN625 [37,38].

**Figure 3.**
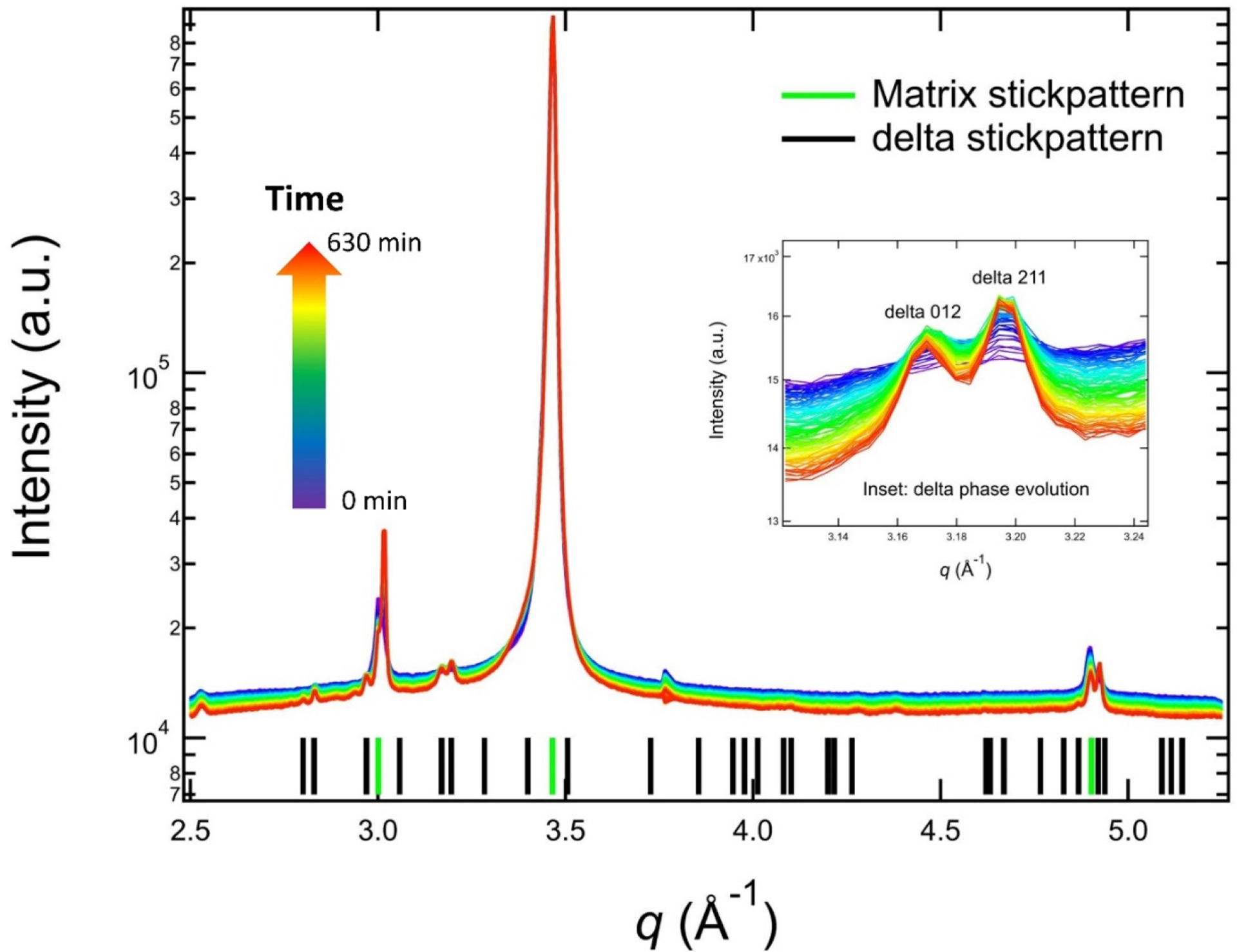
In situ synchrotron XRD data acquired during an isothermal heat treatment of AM IN625 at 700 °C. The inset shows the evolution of the δ 012 peak and δ 211 peak. The data acquisition time follows the arrowed color scale. The calculated stick patterns correspond to an FCC matrix phase and an orthorhombic δ phase.

Figure 3 shows the in situ XRD data of AM IN625 acquired during an isothermal hold at 700 °C for 10.5 h. The XRD data obtained before the heat treatment at room temperature suggest that the IN625 in its as-fabricated state has an FCC matrix phase with a lattice constant of (3.595 ± 0.002) Å, with no additional detectable phases. It is worth noting that the synchrotron XRD measurements were conducted with high flux and highly penetrating X-rays using a single-photon counting detector. This measurement sensitivity means that the equilibrium phases predicted in the phase diagram other than the matrix phase did not have adequate time to form in any significant amount during the build. The single-phase as-fabricated matrix phase represents the starting point of the subsequent solid-state phase transformation.

The in situ XRD data acquired during the heat treatment allow us to monitor the thermally induced phase transformation. As shown in Figure 3, the XRD data continuously evolved at 700 °C, with the main feature being a monotonic increase in peak intensities of a new family of peaks. These new peaks belong to an orthorhombic structure. The stick patterns in Figure 3 are calculated based on an orthorhombic phase with lattice parameters of 5.109 Å, 4.232 Å, and 4.487Å and an FCC phase with a lattice constant of 3.626 Å, respectively. These lattice parameters are the values at 700 °C to directly compare the stick patterns and the in situ experimental data. The δ peaks are weak. Hence, we used an inset to highlight the time-dependent changes of two characteristic peaks of the δ phase (δ 012 and δ 211). In addition to the continuous growth of the peak intensity, we also observed a narrowing of the peak width, which is indicative of precipitate growth.

**Figure 4.**
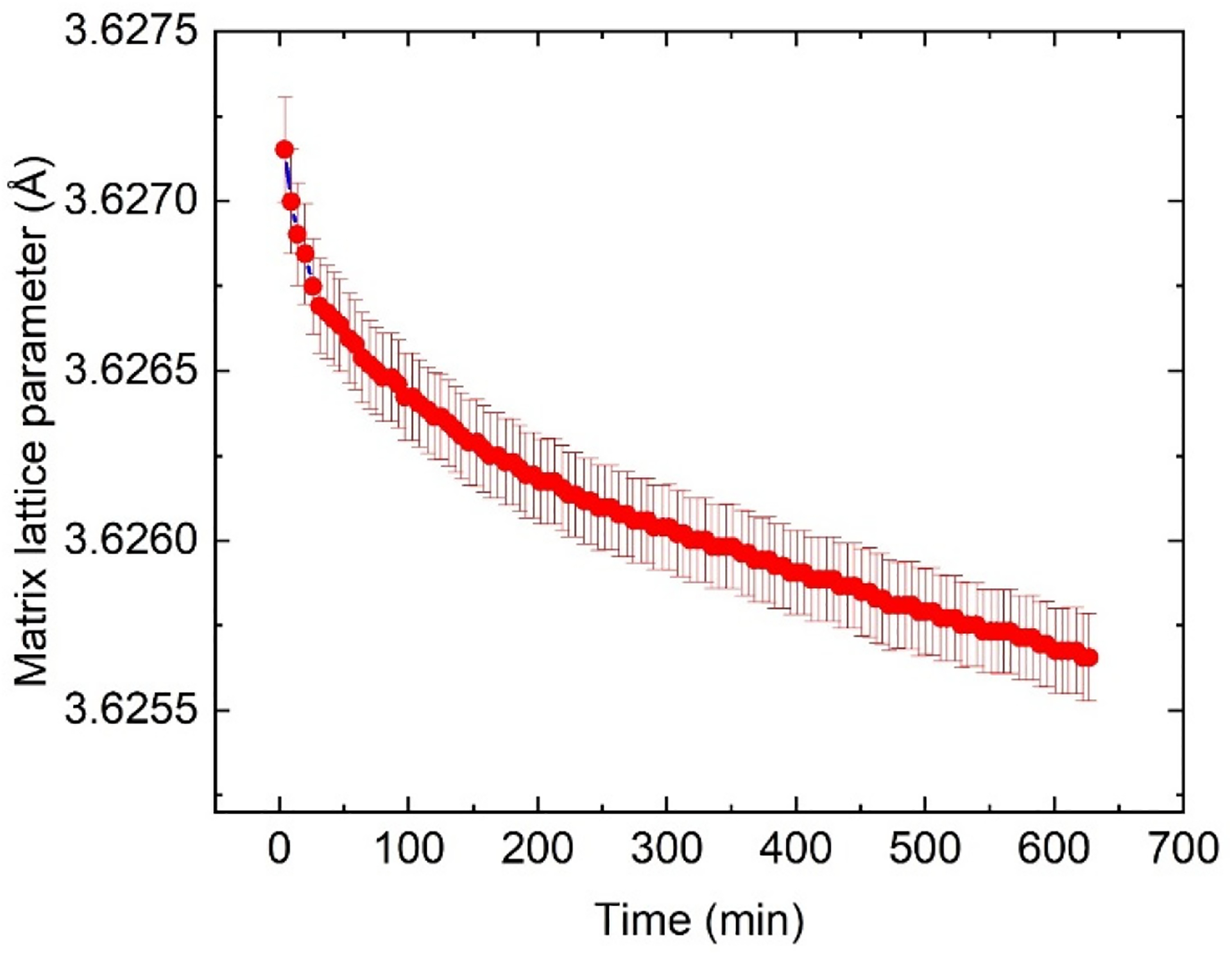
Time dependence of the matrix lattice constant, acquired from the in situ XRD measurements at 700 °C. We observed a monotonic decrease in the lattice parameter, indicating that heavy elements such as Nb and Mo gradually diffuse from the solid-solution matrix and contribute to the nucleation and growth of the δ phase. The uncertainty reported in this figure and hereafter represents one standard deviation unless specified otherwise.

A careful analysis based on the in situ XRD could reveal the structural changes in both the FCC matrix and the precipitates. Figure 4 shows the evolution of the lattice constant of the FCC matrix. We observed a monotonic decrease in the lattice constant, indicating that the elements with large atomic radii, such as Nb and Mo, were gradually depleted from the matrix. This phenomenon is consistent with the precipitation of δ phase precipitates that consume Nb and Mo, as illustrated by Figure 3. This reduction in the matrix lattice parameter associated with the precipitation of the δ phase precipitates is also observed in service-exposed IN625 [39], except that a prolonged heat treatment (500 h) at 850 °C is required for the change in lattice parameter to be detectable.

Additionally, the change in the matrix lattice parameter of a solid-solution alloy is associated with the extent of the precipitation [40]. In AM IN625, the difference in the matrix lattice parameters before and after a 10 h heat treatment at 870 °C was ≈0.0042 Å [21]. In comparison, the lattice parameter changed by ≈0.0015 Å after a 10.5 h heat treatment at 700 °C, suggesting significantly less precipitation of the δ phase at this temperature.

IN625, as designed, is a single-phase alloy where the strength mainly originates from the solid solution strengthening from Mo, Nb, and Cr [1]. Whereas the depletion of Mo and Nb from the matrix is expected to reduce the strength, the formation of precipitates can compensate for this reduction and increase the overall strength of IN625. For example, wrought IN625 reaches its peak hardness after a 170 h heat treatment at 700 °C, which is mainly due to the precipitation of the γ″ phase, a precursor to the δ phase [41]. Similarly, precipitation of the δ phase also increases the overall strength and reduces the ductility [37]. For AM IN625, a systematic evaluation of the effect of heat treatment on mechanical properties over a range of temperatures is required and needed.

**Figure 5.**
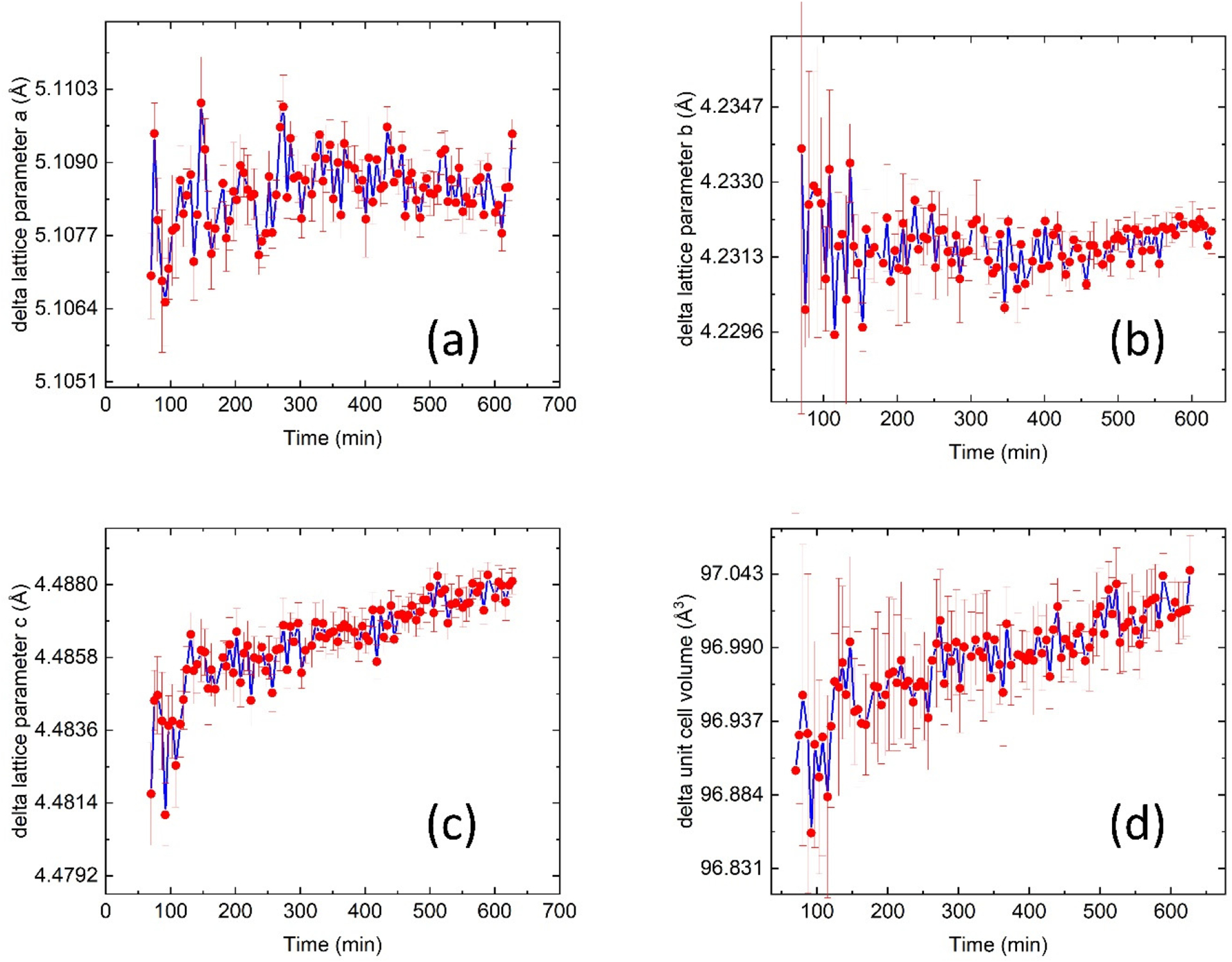
(**a**–**c**) Time dependence of the three lattice parameters of the δ phase precipitates, acquired from the in situ XRD measurements at 700 °C. (**d**) The volume of the unit cell of the δ phase precipitates.

During the heat treatment, the unit cell of the δ phase also changes. Figure 5 illustrates this change. Among the three orthorhombic lattice parameters (Figure 5a–c), two are nearly constant at ≈5.108 Å and ≈4.232 Å, respectively. The third lattice parameter shows a monotonic increase from ≈4.482 to ≈4.488 Å. It is known that the long axis of the δ phase aligns with the close-packed directions of the FCC matrix, and the crystallo-graphic orientations between the FCC matrix and the δ phase follow {111}_FCC_//(100)_δ_ and $<1\bar{1}0{>}_{\text{FCC}}//{\left[100\right]}_{\delta }$ [10]. Based on this, we infer that the diffusion of Nb and Mo is also directional. Since Mo diffusion from the matrix to the δ phase occurs more slowly than that of Nb [42], this directional diffusion may lead to a change in δ phase chemistry and an increase in the unit cell volume, as shown in Figure 5d.

**Figure 6.**
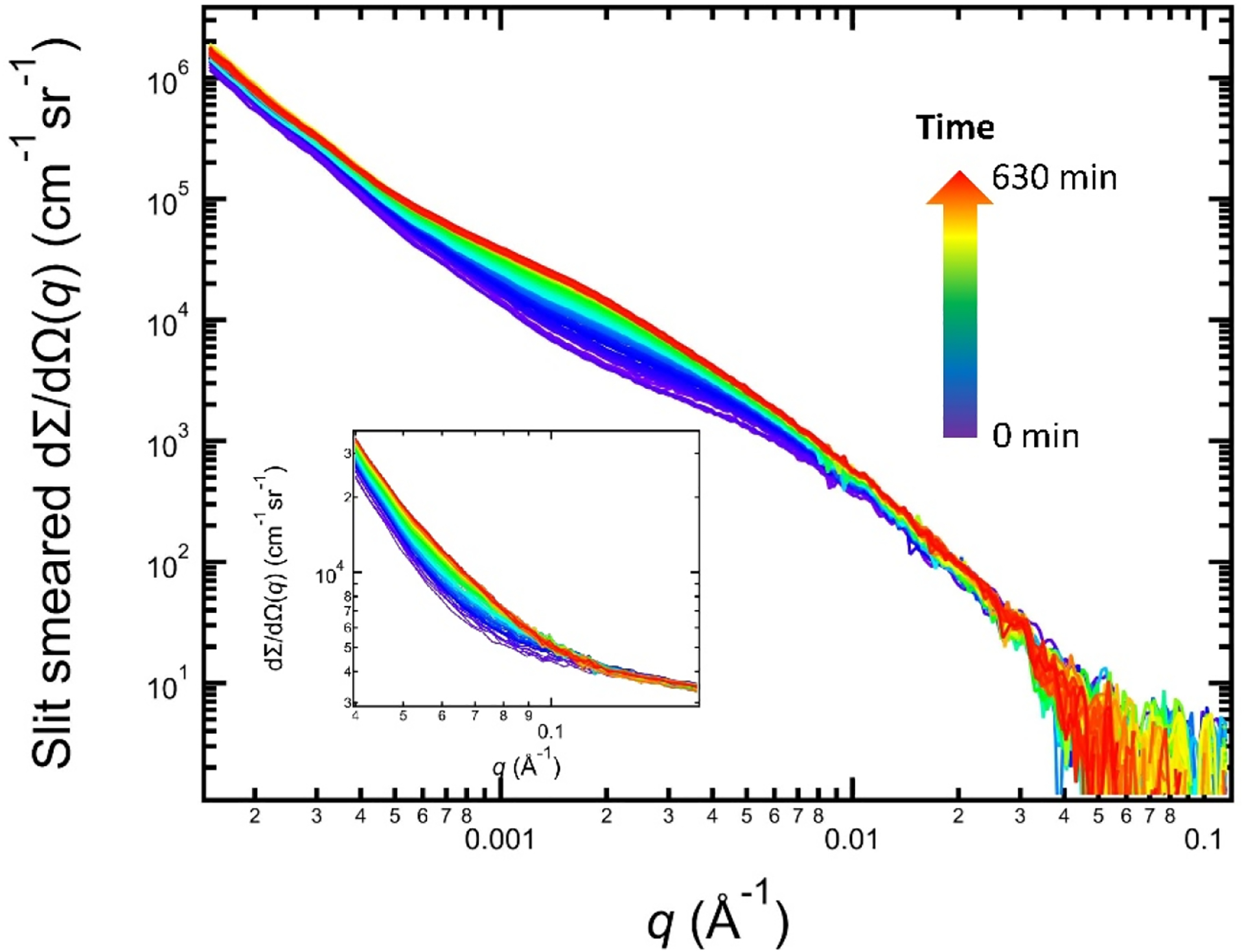
In situ SAXS data of AM IN625 acquired during isothermal heat treatment at 700 °C for 10.5 h. The main plot and the inset show the USAXS and SAXS data, respectively. The acquisition time is color-coded following the time arrow. The USAXS intensity is slit-smeared.

In situ SAXS data acquired with the same sample volume during the isothermal heat treatment also provide a window to probe the statistically significant transformation kinetics of the material’s microstructure. Figure 6 shows the complete dataset, with USAXS data being the main plot and the SAXS data shown in the inset. For consistency, the scattering data are color-coded using the same color scale as the XRD data in Figure 3. The scattering data have three noticeable characteristics. First, for the very low-*q* part of the scattering data (≈1 × 10^−4^ Å^−1^ to ≈4 × 10^−4^ Å^−1^), we observed a power-law slope that does not change as a function of time. We attribute this feature to the grain scattering, which is similar to previous work on Ni-based superalloys [21,43] and aluminum alloys [44,45]. The grain growth in IN625 is minimal for temperatures below 900 °C [46]. Hence, the grain scattering is expected to be stable, which is consistent with experimental observations. Second, we observed a monotonic increase in scattering intensity between ≈4 × 10^−4^ Å^−1^ and ≈0.01 Å^−1^, notably with two Guinier regions near 2 × 10^−3^ Å^−1^ and 8 × 10^−3^ Å^−1^, respectively. Since the in situ XRD data and ex situ SEM images only show the δ phase precipitates, we attribute this scattering signal to the δ phase. Previous microscopic studies have established that the δ phase precipitates are platelets with two characteristic sizes [10,21,47], which is in agreement with the observation of two Guinier regions in the scattering data. Lastly, the SAXS data shown in the inset are simple extensions of the high *q* power-law slope in the USAXS data; i.e., the SAXS data contain no additional information, which indicates that no additional nm-sized precipitates formed during this heat treatment.

**Figure 7.**
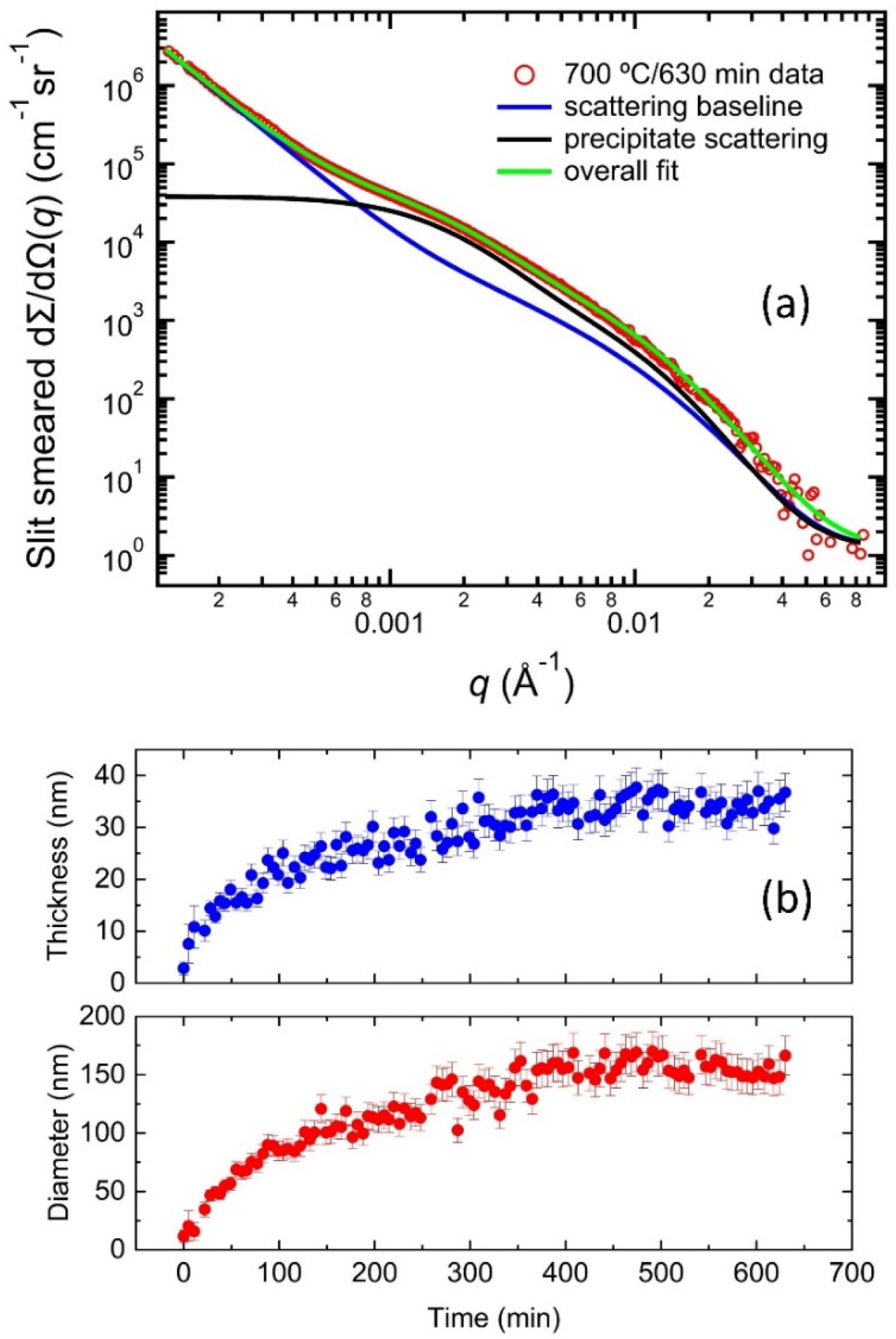
(**a**) An illustration of the SAXS model used in this work. The data were acquired at 630 min during the heat treatment at 700 °C. The overcall scattering comprises two parts: (1) scattering baseline and (2) excess scattering from the δ phase precipitates. (**b**) The time-dependent evolution of the mean diameter (major dimension) and thickness (minor dimension) of the δ phase precipitates.

We constructed a scattering model to describe the scattering data based on these observations, as illustrated in Figure 7a. Using the USAXS data acquired at 630 min into the in situ experiment as an example, this model consists of two components. The first component is the scattering baseline, which is obtained on the same sample volume at room temperature before the heat treatment. The second component represents the excess scattering originating from the δ phase precipitates. As established previously [21], we described this excess scattering using an analysis approach analogous to the unified small-angle scattering method with two scattering levels [48]. Together, this two-component model describes the in situ SAXS data well through the entire data sequence.

Figure 7b shows the evolution of the mean thickness (minor dimension) and diameter (major dimension) of the δ phase precipitates at 700 °C as a function of time. The thickness and diameter demonstrate a similar trend, with an initial rapid increase followed by a gradual increase. By the end of the heat treatment, the mean thickness and diameter are 34 ± 2 nm and 154 ± 7 nm, respectively. These values are significantly smaller than the values acquired from AM IN625 after 10 h at 870 °C, where the mean thickness and diameter are 52 ± 5 nm and 961 ± 94 nm, respectively [21], again pointing to significantly slower precipitation kinetics at 700 °C. In the context of typical residual stress heat treatment, after a one-hour heat treatment at 870 °C, the mean thickness and diameter are 45 ± 4 nm and 424 ± 40 nm, respectively [21]; after a two-hour heat treatment at 800 °C, the mean thickness and diameter, depending on the build condition, range between 61 nm to 77 nm and 416 nm to 634 nm, respectively [24]. In other words, a stress relief heat treatment at 700 °C for as long as 10 h results in δ phase precipitates significantly smaller than those developed during typical residual stress heat treatment of AM 625.

It is worth noting that the continuous coarsening of the δ phase precipitates observed at 870 °C was not apparent at 700 °C, suggesting stability against significant coarsening at 700 °C, which is possibly due to the stabilization provided by the elastic energy of the strain field surrounded by the precipitates [49]. This limited growth of the δ phase precipitates during the long heat treatment at 700 °C is significant because the overgrown δ phase leads to reduced fracture strain [50]. Moreover, a recent review shows that direct aging at 700 °C for 24 h also leads to the highest reported UTS (1222 MPa) and yield strength (1012 MPa) for AM IN625, suggesting that the formation of smaller precipitates serves to improve the mechanical strength [51].

Compared with previously reported kinetics at 800 °C and 870 °C, we observed significantly slower precipitation of the δ phase precipitates at 700 °C in AM IN625. To rationalize our observations, we used thermodynamic calculations to understand the precipitation kinetics.

In our simulations, we assumed that all the precipitates are spherical. We also assumed that the nucleation occurs on dislocations because the pre-existing interface helps reducing the surface energy barrier of nucleation [52]. During AM processing, the compression-tension residual stress cycles induced by the localized, extreme heating and cooling conditions cause a heterogeneous distribution of local dislocation densities [53]. Consistent with previous work [33], we assumed that the dislocation density is ≈5 × 10^11^ m^−2^. This dislocation density corresponds to a nucleation site density of ≈10^21^ m^−3^. For the precipitation simulation, we considered δ, γ^″^, MC carbide, μ, and σ precipitates, with the matrix phase being γ. We assumed the interfacial energies are 20 mJ/m^2^, 55 mJ/m^2^, 60 mJ/m^2^, 200 mJ/m^2^, and 200 mJ/m^2^ for the γ/γ^″^, *γ*/*δ*, γ/MC, *γ*/*μ*, and *γ*/*σ* interfaces, respectively. More details about the simulation can be found elsewhere [33].

**Figure 8.**
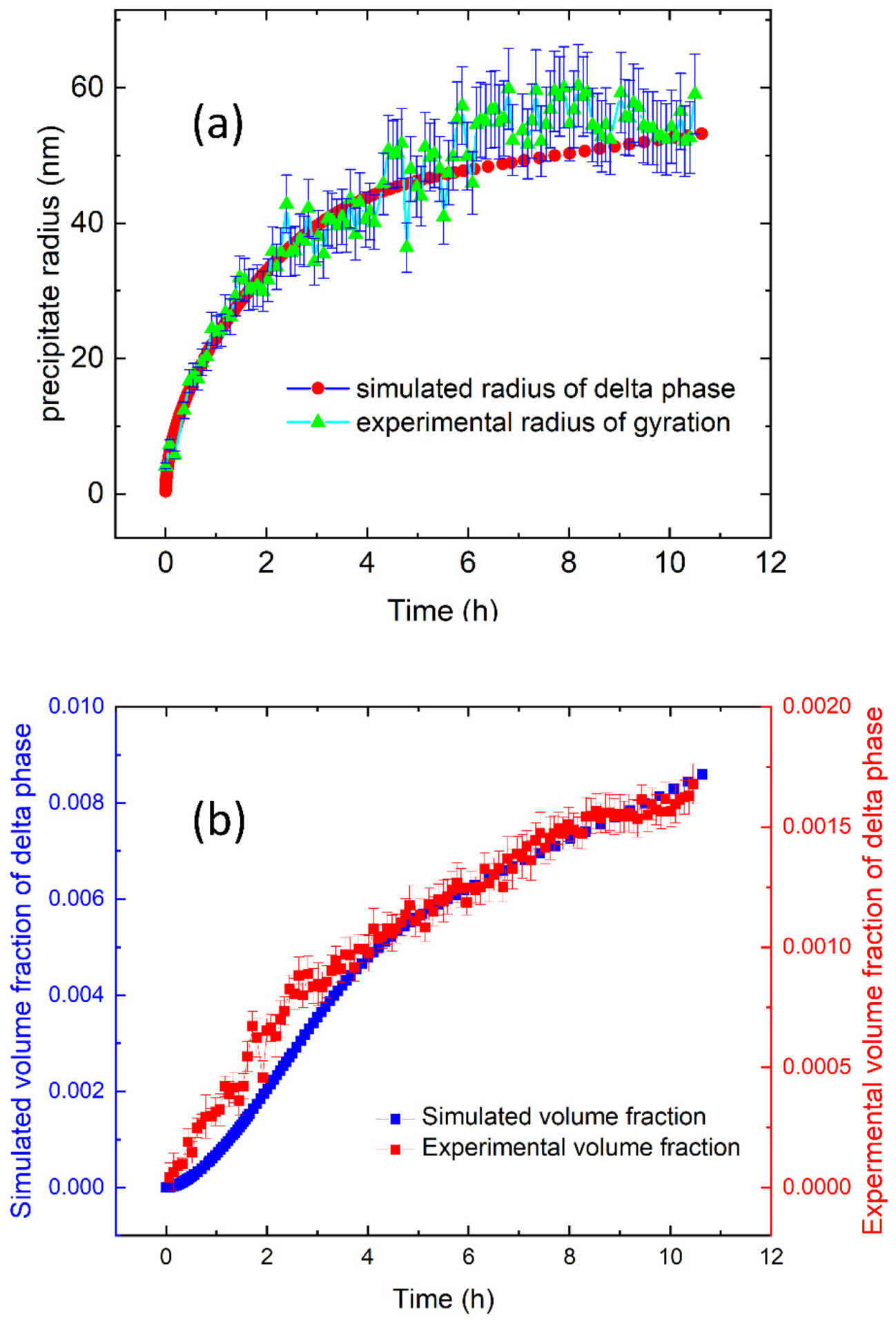
(**a**) A comparison between the calculated (simulated) radius and experimental mean radius of gyration of the δ phase precipitates at 700 °C as a function of annealing time. Here, we assumed a spherical morphology for the precipitates for the simulation. Accordingly, we calculated the radius of gyration of the platelet δ phase precipitates based on experimental values reported in Figure 7b. (**b**) A comparison between the calculated and experimental volume fraction of the δ phase precipitates at 700 °C as a function of time.

As a result of the microsegregation, the composition between the neighboring interdendritic regions is not uniform. Previous SEM measurements have shown that the secondary dendritic arm spacing of the as-fabricated AM IN625 is ≈300 nm [19]. DICTRA simulation shows that microsegregation is confined to ≈20 nm from the interdendritic centers [33]. In other words, the average composition represents a good approximation for a redistributed composition. Figure 8 shows the comparison between the experimental results and the TC-PRISMA predictions with the nominal composition. Since we assume a spherical shape for the precipitates in the simulation, we converted the observed platelet size into a radius of gyration (Rg) for direct comparison following Rg^2^ = R^2^/2 + D^2^/12, where R and D represents one-half of the diameter and the thickness as reported in Figure 7b, respectively. Figure 8a shows that the model-predicted radius and the effective measured Rg follow a similar kinetic trend with the simulated radius slightly smaller than the experimental value, as reflected by the Rg. When we simulate the precipitation reaction with a composition adjusted to the enriched interdendritic region, our simulations predict slightly larger precipitates with a similar kinetic time scale. Hence, a weighted average of the simulated precipitate radii associated with the interdendritic regions and dendrites is expected to be closer to the experimental values. Figure 8b shows that the simulated time-dependent volume fraction and the experimental volume fraction, acquired following a protocol detailed previously, have a similar trend except that the experimental value is smaller by a factor of ≈5. This discrepancy is similar to previously reported results acquired at 800 °C and 870 °C. Several factors could contribute to the quantitative difference, including assumed spherical geometry of the precipitates, dislocation density, and a temperature-dependence of the interfacial energy. Notwithstanding these reservations, our results still represent a good agreement between simulations and experiments given the approximate nature of the simulations.

## 4. Conclusions

In this work, we performed a detailed analysis of the precipitation kinetics of an AM IN625 alloy fabricated using L-PBF during heat treatment at 700 °C. Whereas previously reported residual stress heat treatments at 800 °C and 870 °C can effectively reduce the residual stress, they lead to the formation of large δ phase precipitates at a significant volume fraction and create unfavorable conditions for applications requiring good ductility, fracture toughness, and corrosion resistance. Our ex situ SEM data show that heat treatment at 700 °C leads to significantly slower precipitation of the δ phase precipitation when compared with 800 °C. The in situ synchrotron XRD data show that the δ phase is the only observable precipitating phase at 700 °C. The time-dependent lattice parameters of the FCC matrix and the δ phase show a continuous contraction of the FCC unit cell and a continuous expansion of the δ phase unit cell, which is consistent with a slow diffusion of Nb and Mo from the matrix phase to the δ phase. The in situ SAXS results reveal that the morphological evolution of the δ phase precipitates behaves differently at 700 °C compared to 800 °C and 870 °C. The major dimension of the platelet δ phase precipitates reached a stable value of 154 ± 7 nm after 10.5 h at 700 °C, which is in contrast to a continuously increasing major dimension that reached 961 ± 94 nm after 10 h at 870 °C. In the context of a residual stress heat treatment, a stress relief heat treatment at 700 °C for as long as 10 h results in δ phase precipitates (major dimension ≈150 nm) significantly smaller than those developed during typical residual stress heat treatment of AM 625 (major dimension ≈500 nm after one hour at 870 °C or two hours at 800 °C). We also compared the experimental findings with a TC-PRISMA-based precipitation simulation. The simulation captured the general trend in precipitation kinetics with good agreement between the observed and simulated precipitate size. The simulation overestimates the volume fraction of the precipitates, which is possibly due to factors such as the assumed spherical geometry of the precipitates, the effects of the dislocation density, and any temperature dependence of the interfacial energy. In general, this work unequivocally established significantly slower precipitation kinetics of the δ phase for AM IN625 at 700 °C than at 800 °C or 870 °C, which are temperatures commonly used for residual stress relief, and this work also provides the rigorous microstructural kinetics data required to explore the feasibility of a lower-temperature stress-relief heat treatment for AM IN625.

## Figures and Tables

**Table 1.** Measured composition of the virgin IN625 feedstock powders used in this work as provided by the vendor-supplied data sheet and determined following ASTM E1019 standard as well as the allowable range of composition of IN625. The testing relative uncertainty for elements with mass fraction between 5 and 25% is ±5% of the value, for elements with mass fractions between 0.05% and 4.99% is ±10% of the value, for elements with mass fractions less than 0.049% is ±25% of the value.

	Cr	Mo	Nb	Fe	Ti	Al	Co	Si	Mn	C	Ni
Measured Mass Fraction (%)	20.7	8.83	3.75	0.72	0.35	0.28	0.18	0.13	0.03	0.01	balance
Standard Range of Mass Fraction (%)	20.0–23.0	8.0–10.0	3.15–4.15	5.0 max	0.4 max	0.4 max	1.0 max	0.5 max	0.5 max	0.1 max	Balance

## References

[1] Floreen S; Fuchs GE; Yang WJ The metallurgy of alloy 625. Superalloys 1994, 718, 13–37.

[2] Yangfan W; Xizhang C; Chuanchu S Microstructure and mechanical properties of Inconel 625 fabricated by wire-arc additive manufacturing. Surf. Coatings Technol 2019, 374, 116–123.

[3] Gonzalez J; Mireles J; Stafford S; Perez M; Terrazas C; Wicker R Characterization of Inconel 625 fabricated using powder-bed-based additive manufacturing technologies. J. Mater. Process. Technol 2019, 264, 200–210.

[4] Pleass C; Jothi S Influence of powder characteristics and additive manufacturing process parameters on the microstructure and mechanical behaviour of Inconel 625 fabricated by Selective Laser Melting. Addit. Manuf 2018, 24, 419–431.

[5] Rivera O; Allison P; Jordon J; Rodriguez O; Brewer L; McClelland Z; Whittington W; Francis D; Su J; Martens R; Microstructures and mechanical behavior of Inconel 625 fabricated by solid-state additive manufacturing. Mater. Sci. Eng. A 2017, 694, 1–9.

[6] Wang Z; Denlinger E; Michaleris P; Stoica AD; Ma D; Beese A Residual stress mapping in Inconel 625 fabricated through additive manufacturing: Method for neutron diffraction measurements to validate thermomechanical model predictions. Mater. Des 2017, 113, 169–177.

[7] List F; Dehoff R; Lowe L; Sames W Properties of Inconel 625 mesh structures grown by electron beam additive manufacturing. Mater. Sci. Eng. A 2014, 615, 191–197.

[8] Martin JH; Yahata BD; Hundley JM; Mayer J; Schaedler TA; Pollock TM 3D printing of high-strength aluminium alloys. Nature 2017, 549, 365–369. 28933439 10.1038/nature23894

[9] DebRoy T; Wei HL; Zuback JS; Mukherjee T; Elmer JW; Milewski JO; Beese AM; Wilson-Heid A; De A; Zhang W Additive manufacturing of metallic components—Process, structure and properties. Prog. Mater. Sci 2018, 92, 112–224.

[10] Lass EA; Stoudt MR; Williams ME; Katz MB; Levine LE; Phan TQ; Gnaeupel-Herold TH; Ng DS Formation of the Ni3Nb δ-Phase in Stress-Relieved Inconel 625 Produced via Laser Powder-Bed Fusion Additive Manufacturing. Met. Mater. Trans. A 2017, 48, 5547–5558. 10.1007/s11661-017-4304-6 PMC11523210 39479225

[11] Megahed M; Mindt H-W; N’Dri N; Duan H; Desmaison O Metal additive-manufacturing process and residual stress modeling. Integr. Mater. Manuf. Innov 2016, 5, 61–93.

[12] Li C; Liu Z; Fang X; Guo Y Residual Stress in Metal Additive Manufacturing. Procedia CIRP 2018, 71, 348–353.

[13] Sun L; Ren X; He J; Zhang Z Numerical investigation of a novel pattern for reducing residual stress in metal additive manufacturing. J. Mater. Sci. Technol 2021, 67, 11–22.

[14] Promoppatum P; Yao S-C Influence of scanning length and energy input on residual stress reduction in metal additive manufacturing: Numerical and experimental studies. J. Manuf. Process 2020, 49, 247–259.

[15] Papadakis L; Chantzis D; Salonitis K On the energy efficiency of pre-heating methods in SLM/SLS processes. Int. J. Adv. Manuf. Technol 2018, 95, 1325–1338.

[16] Ghosh S; Ma L; Ofori-Opoku N; Guyer JE On the primary spacing and microsegregation of cellular dendrites in laser deposited Ni–Nb alloys. Model. Simul. Mater. Sci. Eng 2017, 25, 065002. 10.1088/1361-651x/aa7369 PMC11497217 39444581

[17] Wang X; Liu PW; Ji Y; Liu Y; Horstemeyer MH; Chen L Investigation on Microsegregation of IN718 Alloy During Additive Manufacturing via Integrated Phase-Field and Finite-Element Modeling. J. Mater. Eng. Perform 2019, 28, 657–665.

[18] Flemings MC Our Understanding of Macrosegregation. Past and Present. ISIJ Int. 2000, 40, 833–841.

[19] Keller T; Lindwall G; Ghosh S; Ma L; Lane BM; Zhang F; Kattner UR; Lass EA; Heigel JC; Idell Y Application of finite element, phase-field, and calphad-based methods to additive manufacturing of ni-based superalloys. Acta Mater. 2017, 139, 244–253. 29230094 10.1016/j.actamat.2017.05.003 PMC5721357

[20] Silva CC; Miranda H; Motta MF; Farias JP; Afonso CRM; Ramirez AJ New insight on the solidification path of an alloy 625 weld overlay. J. Mater. Res. Technol 2013, 2, 228–237.

[21] Zhang F; Levine LE; Allen AJ; Stoudt MR; Lindwall G; Lass EA; Williams ME; Idell Y; Campbell CE Effect of heat treatment on the microstructural evolution of a nickel-based superalloy additive-manufactured by laser powder bed fusion. Acta Mater. 2018, 152, 200–214. 10.1016/j.actamat.2018.03.017 PMC6508661 31080354

[22] EOS NickeAlloy IN625. Available online: https://www.eos.info/03_system-related-assets/material-related-contents/metal-materials-and-examples/metal-material-datasheet/nickelalloy-inconel/niall-in625-m290_material_data_sheet_06-17_en.pdf (accessed on 13 August 2021).

[23] Ilavsky J; Zhang F; Andrews RN; Kuzmenko I; Jemian PR; Levine LE; Allen AJ Development of combined microstructure and structure characterization facility for in situ and operando studies at the Advanced Photon Source. J. Appl. Crystallogr 2018, 51, 867–882. 10.1107/S160057671800643X PMC6463311 30996401

[24] Zhang F; Levine LE; Allen AJ; Young SW; Williams ME; Stoudt MR; Moon K-W; Heigel JC; Ilavsky J Phase Fraction and Evolution of Additively Manufactured (AM) 15–5 Stainless Steel and Inconel 625 AM-Bench Artifacts. Integr. Mater. Manuf. Innov 2019, 8, 362–377. 10.1007/s40192-019-00148-1 PMC7067001 32166056

[25] Andersson J-O; Helander T; Höglund L; Shi P; Sundman B Thermo-Calc & DICTRA, computational tools for materials science. Calphad 2002, 26, 273–312.

[26] Software, T.-C. Ni-Based Superalloys Database, Version 8. Available online: http://www.thermocalc.com/products-services/databases/thermodynamic/ (accessed on 1 September 2015).

[27] Tc-Prisma Version 2.0.3; Thermo-Calc Software Ab: Stockholm, Sweden, 2013.

[28] Shi PF; Engström A; Sundman B; Ågren J Thermodynamic Calculations and Kinetic Simulations of some Advanced Materials. Mater. Sci. Forum 2011, 675–677, 961–974.

[29] Chen Q; Wu K; Sterner G; Mason P Modeling precipitation kinetics during heat treatment with calphad-based tools. J. Mater. Eng. Perform 2014, 23, 4193–4196.

[30] Langer JS; Schwartz AJ Kinetics of nucleation in near-critical fluids. Phys. Rev. A 1980, 21, 948–958.

[31] Kampmann R; Wagner R Decomposition of Alloys: The early stages. In Proceedings of the 2nd Acta-Scripta Metallurgica Conference, Sonnenberg, Germany, 19–23 September 1983, Pergamon Press: Oxford, UK, 1984; pp. 91–103.

[32] Wagner R; Kampmann R; Voorhees PW Homogeneous second-phase precipitation. Mater. Sci. Technol 1991, 5, 309.

[33] Lindwall G; Campbell CE; Lass E; Zhang F; Stoudt MR; Allen AJ; Levine LE Simulation of TTT Curves for Additively Manufactured Inconel 625. Met. Mater. Trans. A 2018, 50, 457–467. 10.1007/s11661-018-4959-7 PMC9706688 36452270

[34] Kirkwood D Microsegregation. Mater. Sci. Eng 1984, 65, 101–109.

[35] Zhang F; Levine LE; Allen AJ; Campbell CE; Lass E; Cheruvathur S; Stoudt MR; Williams ME; Idell Y Homogenization kinetics of a nickel-based superalloy produced by powder bed fusion laser sintering. Scr. Mater 2017, 131, 98–102. 28824284 10.1016/j.scriptamat.2016.12.037 PMC5557300

[36] Stoudt MR; Lass E; Ng DS; Williams ME; Zhang F; Campbell CE; Lindwall G; Levine LE The Influence of Annealing Temperature and Time on the Formation of δ-Phase in Additively-Manufactured Inconel 625. Met. Mater. Trans. A 2018, 49, 3028–3037. 10.1007/s11661-018-4643-y PMC6459739 30983847

[37] Shankar V; Rao KBS; Mannan S Microstructure and mechanical properties of Inconel 625 superalloy. J. Nucl. Mater 2001, 288, 222–232.

[38] Sundararaman M; Mukhopadhyay P; Banerjee S Precipitation of the δ-Ni3Nb phase in two nickel base superalloys. Met. Mater. Trans. A 1988, 19, 453–465.

[39] Rai SK; Kumar A; Shankar V; Jayakumar T; Rao KBS; Raj B Characterization of microstructures in Inconel 625 using X-ray diffraction peak broadening and lattice parameter measurements. Scr. Mater 2004, 51, 59–63.

[40] Cocks FH A lattice parameter method for the investigation of solid state precipitation. J. Mater. Sci 1972, 7, 771–780.

[41] Shaikh M; Ahmad M; Shoaib K; Akhter J; Iqbal M Precipitation hardening in Inconel*625. Mater. Sci. Technol 2000, 16, 129–132.

[42] Karunaratne M; Reed R Interdiffusion of Niobium and Molybdenum in Nickel between 900–1300 °C. Defect and Diffusion Forum. 2005, 237–240, 420–425.

[43] Andrews RN; Serio J; Muralidharan G; Ilavsky J An in situ usaxs–saxs–waxs study of precipitate size distribution evolution in a model ni-based alloy. J. Appl. Crystallogr 2017, 50, 734–740. 28656039 10.1107/S1600576717006446 PMC5458593

[44] Jia Q; Zhang F; Rometsch P; Li J; Mata J; Weyland M; Bourgeois L; Sui M; Wu X Precipitation kinetics, microstructure evolution and mechanical behavior of a developed Al–Mn–Sc alloy fabricated by selective laser melting. Acta Mater. 2020, 193, 239–251. PMC7574377 33093793

[45] Zhang F; Levine LE; Allen AJ; Campbell CE; Creuziger AA; Kazantseva N; Ilavsky J In situ structural characterization of ageing kinetics in aluminum alloy 2024 across angstrom-to-micrometer length scales. Acta Mater. 2016, 111, 385–398. 29606898 10.1016/j.actamat.2016.03.058 PMC5876935

[46] Liu M; Zheng W-J; Xiang J-Z; Song Z-G; Pu E-X; Feng H Grain Growth Behavior of Inconel 625 Superalloy. J. Iron Steel Res. Int 2016, 23, 1111–1118.

[47] Qin S; Novak TC; Vailhe MK; Liu Z-K; Beese AM Plasticity and fracture behavior of Inconel 625 manufactured by laser powder bed fusion: Comparison between as-built and stress relieved conditions. Mater. Sci. Eng. A 2021, 806, 140808.

[48] Beaucage G Approximations Leading to a Unified Exponential/Power-Law Approach to Small-Angle Scattering. J. Appl. Crystallogr 1995, 28, 717–728.

[49] Johnson WC; Alexander JID Interfacial conditions for thermomechanical equilibrium in two-phase crystals. J. Appl. Phys 1986, 59, 2735–2746.

[50] Liu D; Zhang X; Qin X; Ding Y High-temperature mechanical properties of Inconel-625: Role of carbides and delta phase. Mater. Sci. Technol 2017, 33, 1610–1617.

[51] Soni H; Gor M; Rajput GS; Sahlot P A comprehensive review on effect of process parameters and heat treatment on tensile strength of additively manufactured Inconel-625. Mater. Today Proc 2021.

[52] Verhoeven JD Fundamentals of Physical Metallurgy; Wiley: New York, NY, USA, 1975.

[53] Wang G; Ouyang H; Fan C; Guo Q; Li Z; Yan W; Li Z The origin of high-density dislocations in additively manufactured metals. Mater. Res. Lett 2020, 8, 283–290.

